# Chronic jet lag reduces motivation and affects other mood-related behaviors in male mice

**DOI:** 10.3389/fphys.2023.1225134

**Published:** 2023-09-06

**Authors:** Julieta Acosta, Manuel T. Crespo, Santiago A. Plano, Diego A. Golombek, Juan J. Chiesa, Patricia V. Agostino

**Affiliations:** ^1^ Department of Science and Technology, Universidad Nacional de Quilmes/CONICET, Buenos Aires, Argentina; ^2^ Institute for Biomedical Research (BIOMED), Universidad Católica Argentina (UCA)/CONICET, Buenos Aires, Argentina; ^3^ Laboratorio Interdisciplinario del Tiempo (LITERA), Universidad de San Andrés/CONICET, Buenos Aires, Argentina

**Keywords:** circadian system, chronic jet lag, depression, motivation, mood-related behaviors

## Abstract

**Introduction:** The circadian system regulates various physiological processes such as sleep-wake cycles, hormone secretion, metabolism, and the reaction to both natural and drug-based rewards. Chronic disruption of the circadian system caused by unsteady synchronization with light-dark (LD) schedules, such as advancing chronic jet lag (CJL), leads to adverse physiological effects and pathologies, and is linked with changes in mood and depressive behaviors in humans and rodent models.

**Methods:** C57BL/6J male mice were subjected to circadian disruption through phase advances of 6 h every 2 days (CJL +6/2). Mice under 12:12-h LD cycle were used as controls. After 8 weeks under these conditions, a battery of behavioral tests was performed to assess if mood-related behaviors were affected.

**Results:** Compared to controls under 24 h LD cycles, mice under CJL presented desynchronization of activity-rest rhythms that led to several behavioral impairments, including a decrease in motivation for food reward, and an increase in anxiety, anhedonia, and depressive-like behavior.

**Conclusion:** Chronic circadian disruption, caused by an experimental CJL protocol, affects mood-related and reward-related behaviors in mice. Understanding the importance of the circadian system and its potential role for disruption due to CJL is important for maintaining good health and well-being.

## Introduction

Daily environmental cycles, such as the alternation between day and night, impose diurnal rhythms in almost all the physiological processes of living organisms. These endogenous self-sustained rhythms are generated by the circadian system, a complex network of peripheral clocks located in different tissues, coordinated by a master oscillator, that helps the organism to predict environmental changes and adapt the physiology and behavior to maintain the organism’s homeostasis Panda et al. (2002); Kim et al. (2019). This master clock is located, in mammals, in the hypothalamic suprachiasmatic nuclei (SCN). The SCN receives direct input from the retina through the retinohypothalamic tract. This pathway provides information to the SCN from the external light/dark cycle, which is its main synchronizer (Golombek and Rosenstein, 2010; Foster et al., 2020).

The misalignment between the circadian system and the light/dark schedule is related to excessive fatigue, decreased cognition and mood, gastrointestinal despair, and diminished psychomotor coordination. Chronic circadian disruption, such as the case of chronic jet lag (CJL), increases the risk for several pathologies, including cancer, metabolic alterations leading to diabetes and obesity, metabolic syndrome, cardiovascular diseases, and premature mortality (Davidson et al., 2006; Maywood et al., 2006; Golombek et al., 2013; Aiello et al., 2020; Horsey et al., 2020; Trebucq et al., 2023). Indeed, CJL can be seen as a shift work model in which individuals experience resynchronization to light/dark cycles for extended periods. The chronic misalignment between the endogenous rhythms and external cycles increases the risk for multiple negative health consequences such as type 2 diabetes, cardiovascular diseases, a defective immune response, cancer, and an augmented risk for neurological diseases (i.e., migraine, epilepsy, and dementia). Moreover, the shift work schedule generates sleep disorders, increased cognitive deficiencies, mood alteration, and mental illnesses such as depression, alcohol abuse, anxiety, and even schizophrenia (Gao et al., 2020).

Recent studies demonstrate that chronic perturbations of circadian rhythms might cause depressive-like behaviors in mice models (Chen et al., 2021). Indeed, persistent irregularities in the patterns of wake and sleep caused by changes in the LD cycle interfere with the correct regulation of the circadian system, and could affect brain areas that regulate motivation and reward-related behaviors, like the nucleus accumbens, the ventral tegmental area, the prefrontal cortex and the amygdala (Lamont, et al., 2005; Acosta, et al., 2020; Chen et al., 2021).

In a previous study, we proved that mice exhibit a consistent circadian pattern of motivation for food reward. Interestingly, this rhythm remained unaffected by the process of aging (Acosta et al., 2020). In the present work, we use a chronic jet lag (CJL) +6/2 light schedule, which consists of a 6-h phase advance of the LD schedule every 2 days, to force a desynchronized activity-rest rhythm (Casiraghi, et al., 2012). This research aims to assess whether CJL causes irregularities in behaviors that depend on motivation and reward processes since the brain areas that regulate mood-related behaviors are under circadian control (McClung, 2007). Understanding the neurological and behavioral mechanisms underlying these phenomena can lead to better treatments for sleep disorders and addiction, as well as improved work schedules and travel policies.

## Materials and methods

### Animals

C57BL/6j male mice, aged 3–4 months, were purchased from the supplier at School of Veterinary Sciences, Universidad Nacional de La Plata, Buenos Aires, Argentina. Animals were initially placed in a controlled environment with a 12:12-h light-dark (LD) cycle, where the lights were turned on at 0800 h. The room temperature was maintained at 20°C ± 2°C, and they had *ad libitum* access to food and water. This acclimation period lasted for a minimum of 2 weeks before subjecting the animals to experimental conditions. During the experiments, whenever mice required handling in dark conditions, a dim red-light source (<5 lux) was used.

The following experiments were approved by the Institutional Animal Care and Use Committee of the University of Quilmes (Buenos Aires, Argentina), and performed in strict accordance with NIH rules for animal care and maintenance.

### Locomotor activity recording

Upon initiation of experimental conditions, mice were moved to individual cages. Each cage was equipped with an infrared sensor, positioned in the top ceiling of the cage, to monitor overall locomotor activity. At the cage level, average light intensity was 200 lux. The activity counts for each mouse were measured by quantifying the total number of infrared sensor beam breaks, and these counts were recorded at 5-min intervals for subsequent analysis (Archron, Buenos Aires, Argentina).

### Chronic jet lag (CJL) model

**FIGURE 1:**
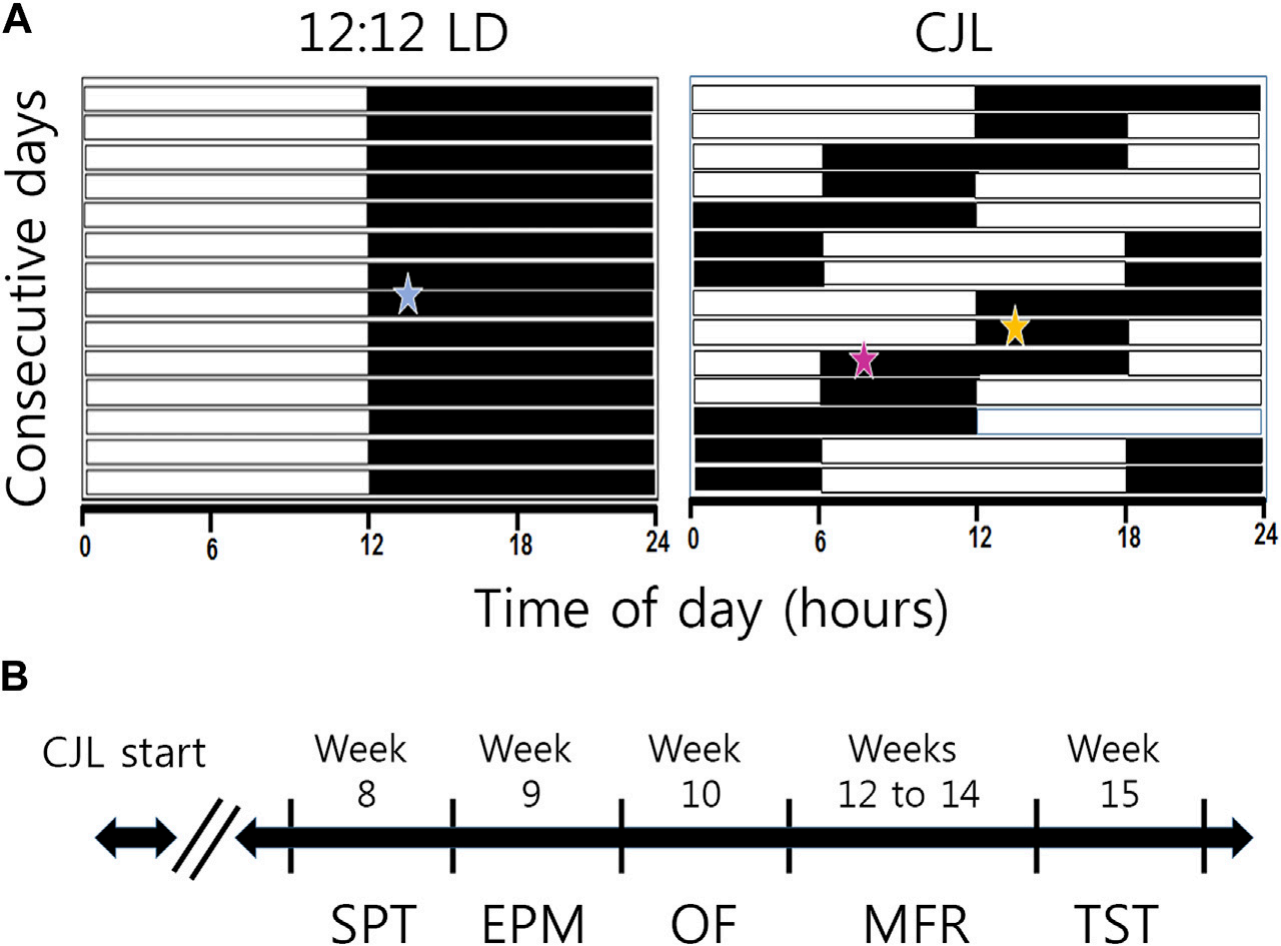
Schematic illustration of experimental groups and timeline. **(A)** Male mice were randomly assigned into control (LD, 12:12 light/dark cycle, N = 8) or chronic jet lag of 6 h advances of the LD cycle every 2 days (CJL +6/2, N = 12) groups. In this kind of chronic phase advances, animals alternate between two nights, a long night of 12 h and a short one of 6 h. Behavioral experiments were conducted during the night, 2 h after lights OFF, as indicated by a blue star in the LD group. For the CJL group, performance during the long night (CJL-LN, pink star) and the short night (CJL-SN, orange star) were compared. **(B)** Behavioral experiments started at week 8 of CJL. Mice were evaluated in the following order: sucrose preference test (SPT, week 8), elevated plus maze (EPM, week 9), open field (OF, week 10), motivation for food reward (MFR, weeks 12–14), and tail suspension test (TST, week 15).

To induce chronic jet lag by advances (ChrA^6/2^), mice were exposed to a schedule of 6-h advances of the LD cycle every 2 days (+6/2). This was achieved by reducing the duration of every second dark phase by 6 h (Figure 1A). Consequently, mice went through a pattern of alternating “short-nights” (only 6 h of darkness) and “long-nights” (12 h of darkness). This chronic jet lag model with the alternating short and long nights was originally reproduced from an already published protocol (Filipski et al., 2004). Our group has previously reported that this protocol of ChrA induces forced desynchronization of locomotor activity rhythms (Casiraghi et al., 2012), altered metabolism (Casiraghi et al., 2016) and tumorigenesis (Aiello et al., 2020) in mice. The control group was housed under 12:12-h LD cycles throughout the experiments, while being subjected to identical conditions to the CJL group in all other aspects.

### Body weight and food intake

Body weight and food intake were monitored once per week until the beginning of behavioral studies (week 8 of CJL, see Figure 1B). Mice were not disrupted for body weight measuring during behavioral experiments except when required (e.g., for caloric restriction during motivation).

### Behavioral experiments

After 8 weeks under CJL, behavioral experiments were assessed. The timeline for the behavioral test battery is shown in Figure 1B. As we have already described a circadian rhythm in motivation (Acosta et al., 2020), all behavioral tests in the present study (except for sucrose preference, which was 24 h long) were conducted during the night at Zeitgeber time (ZT) 14, being ZT 12 the time of lights OFF. That is, the behavioral tests were performed 2 h after lights OFF in both CJL and control (LD) groups. Time of lights OFF was always 19:00 for the LD group, and behavioral experiments began at 21:00 h. Under the CJL protocol, time of lights OFF varied according to the 6 h phase advance, so the beginning of behavioral experiments also varied to meet the criterion of 2 h after lights OFF (see Figure 1A; Supplementary Figure S2). Given that the CJL group experiences two different types of nights throughout the experimental protocol—in terms of the duration of hours of darkness due to phase advances—we decided to subdivide this group into two subgroups: CJL short night (CJL-SN, 6 h of dark exposure) and CJL long night (CJL-LN, 12 h of dark exposure, see Figure 1A) and evaluated them in the behavioral tests separately. Thus, the group naming under CJL refers to the type of protocol used (i.e., a ChrA^6/2^ protocol with a long night and a short night).

Animals were carried to the experimental room 10 min before for habituation. The entire set of behavioral tests were conducted under low-intensity red light (<5 lux) to minimize the impact of light exposure during the nighttime phase.

### Motivation for food reward

The equipment consisted of 4 matching lever boxes for mice (Model ENV-307A, Med Associates, St. Albans, VT) located inside of sound-attenuating chambers (Model ENV-021M; Med Associates). The dimensions of each lever box were 21.59 × 17.78 × 12.70 cm. Each box was constructed with clear Plexiglas for the ceiling, side walls, and door. The front and back walls were made of stainless-steel panels, while the floor consisted of parallel stainless-steel bars. On the front wall of each box, there were retractable levers positioned on the left and right sides, with a food cup placed in between. A pellet dispenser was used to deliver food rewards into the food cup. Rewards consisted of 20-mg grain-based food pellets (cat num F0163, Bio-Serv, Frenchtown, NJ). The operant chambers were managed by the Med-PC IV software package. The fan within each cage remained active throughout the entire session. The house light inside each cage was maintained OFF during testing. A computer connected to an electronic interface (MED Associates, Inc., model DIG-700 and SG-215) was used to control the equipment and register the data. The timing of each lever press was recorded with a precision of 10 milliseconds and placed into 1-s time bins.

The progressive ratio (PR) training was performed to evaluate the level of effort a mouse was disposed to exert in order to obtain a reward (Richardson and Roberts, 1996; Drew et al., 2007; DePoy et al., 2017; Acosta et al., 2020). Seven days before the PR test, mice were calorie restricted to keep them at 85%–90% of their free-feeding weight. This allowed mice to be eager to do work (press the lever) to obtain food pellets as rewards during the task. Mice were evaluated in two consecutive phases: 1) operant lever press training, and 2) progressive ratio (PR) schedule, as previously described (Acosta et al., 2020). In all cases, mice were weighed prior to each session and home-cage food was supplied after sessions.

1) *Operant lever-press training*: Each mouse underwent a daily session of lever-press training for three consecutive days. During the session, a single lever—either left or right, with equal distribution among subjects—was presented. Pressing the lever resulted in the delivery of a food pellet. Sessions concluded either after the mouse received 60 food pellets or 60 min elapsed, whichever came first. Acquisition criterion for operant lever press training was 75% of total lever presses in the last training session. All animals except one from the CJL-SN group—which was excluded from further analysis of motivation behavior—met this criterion.
2) *Progressive ratio (PR) schedule.* After successfully completing the operant lever-press training, mice underwent evaluation in the PR paradigm. They received one PR session over two consecutive days. Data analysis focused only on the results obtained from the second PR session. The PR schedule used has been previously described elsewhere (Richardson and Roberts, 1996; Ramkisoensing and Meijer, 2015; Acosta et al., 2020). Briefly, at the start of each session, a single lever was presented, and the reward was given only after the mouse had met a specific criterion for the number of lever presses needed to obtain the reward. The criterion number of lever presses needed in each trial within a session was obtained from the following equation:

$$P=\left[5\times e^{\left(i\times 0.2\right)}\right]-5$$

where *P* is the required number of lever presses (rounded to the nearest integer) and *i* refers to the trial number. This equation results in the following arithmetic series: 1, 2, 4, 9, 12, 15, 20, 25, 32, 40, 50, 62, 77, 95, 118, 145.178, 219, 268, 328, 402, 492, 603, 737, 901, 1102, 1347, 1647, 2012, etc. Consequently, at the beginning of each session, mice were required to perform one lever press to receive the food reward in the initial trial. Subsequently, for the second trial, they needed to complete two lever presses, followed by four lever presses for the third trial, and so on, increasing progressively according to the equation mentioned. The session ended after 2 h or after 10 min had elapsed without the mouse performing a lever press. Motivation was measured by recording the total number of lever presses made within the session, the total number of rewards earned, the breaking point, and the percentage of animals that actively maintained lever-pressing throughout the duration of the PR session (survival %). The “breaking point” was defined as the last successful criterion the animal was able to complete, from which it received a reward.

### Sucrose preference test

The sucrose preference test was used to evaluate anhedonia according to a modified version of a previously published protocol (Sáenz et al., 2006). Two bottles with 100 mL of either 2% sucrose solution or tap water were placed in each mouse’s home cage at the beginning of the dark phase. The bottles were left in the cage for mice to freely access to them for 24 h. The volume of the liquid was measured before and after the tests to calculate sucrose preference as a percentage of total liquid consumption following the equation:

$$\frac{sucrose\;consumption}{total\;liquid\;consumption}x\;100$$

### Open field test

Exploratory and anxious behavior were assessed by allowing the animals to freely explore a novel open field arena. The arena consisted of a rectangular area of 30 × 50 cm surrounded by a 15 cm high wall located in a room with dim red light <5 lux. The mouse was placed in the center of the arena and its activity during the subsequent 5 min was recorded using a camera mounted on the ceiling. Time spent in the center, periphery and corners as well as total distance traveled, were analyzed using video-tracking software (ANY-maze®, Stoelting CO, United States). Test arenas were cleaned with a non-odor detergent after each mouse.

### Elevated plus maze test

To examine anxiety-like behavior, mice were evaluated in the elevated plus maze. The maze consisted of two open and two closed arms (50 × 10 × 40 cm) made of wood, elevated 50 cm above the floor. The illumination level was dim red light <5 lux. At the beginning of the test, the mouse was placed on the central platform of the apparatus facing one of the closed arms. The number of entries into the open arms and the time the animal spent exploring the open and closed arms were recorded for 5 min using a camera mounted on the ceiling. An arm entry was defined as all four feet in the arm. Data were analyzed using video-tracking software (ANY-maze®, Stoelting CO, United States). The maze was cleaned after each trial with a non-odor detergent.

### Tail suspension test

Behavioral despair was assessed using the tail suspension test. Mice were firmly attached to a flat surface using adhesive tape applied to the tip of their tails, following the method described by Stéru et al., 1987. The animals were suspended approximately 30 cm below the surface and their activity was recorded by camera for 6 min. The illumination level was dim red light <5 lux. The total time of immobility for each mouse was manually scored throughout the 6-min testing session. Immobility was defined as the state in which the animal hung passively without any limb movement.

### Data analysis

Locomotor activity data were analyzed to detect significant periods under LD or CJL by using Bonferroni-corrected Sokolove-Bushell (SB) periodograms, covering a range from 20 to 27 h (Casiraghi et al., 2012). SB periodograms were performed with El Temps software (A. Díez-Noguera, Barcelona, Spain). Behavioral experiments comparing LD controls, CJL-LN, and CJL-SN were analyzed through one-way ANOVA followed by Tukey’s comparison test. Homogeneity of variances and normal distributions were tested using the Brown-Forsythe test and the Shapiro-Wilk test, respectively. The ROUT method (with Q set to 1%) was used to identify outliers. The nonparametric Kruskal–Wallis one-way ANOVA was used when data did not meet assumptions of normality. Body weight and food intake across time were analyzed by using two-way repeated measures ANOVA. For motivation, performance in the progressive ratio schedule was evaluated through the Kaplan-Meier survival function (Drew et al., 2007; Acosta et al., 2020). A log-rank (Mantel-Cox) test was used to determine survival differences between groups. Statistical analyses were performed using GraphPad Prism 8 (GraphPad Software Inc., CA, United States). In all cases, the alpha probability level for type I error was set at *p* = 0.05.

## Results

SB periodogram analysis of locomotor activity recording indicated that mice were behaviorally desynchronized after 8 weeks of CJL, as indicated by the presence of a significant short period component equal to 1260 min (21 h), following the LD schedule, and a significant long period component of average 1511 ± 6.5 min (25.2 ± 0.11 h, mean ± S.D.), as previously reported (Casiraghi et al., 2012). Supplementary Figures 1A,B show representative actograms with their respective periodograms for LD and CJL animals, respectively. Supplementary Table S1 displays the short and long period components for the 12 mice under CJL evaluated, while Supplementary Figure S2 shows the short and long period components of 4 representative animals under CJL.

The analysis of body weight and daily food intake shows no differences for CJL and LD groups (body weight: *p* = 0.6633; daily food intake: *p* = 0.7428, two-way repeated measures ANOVA). Although the CJL group tended to eat less amount of food and to weigh the same as the LD controls, this difference was not statistically significant (see Supplementary Figure S3).

### CJL decreased motivation for food reward

**FIGURE 2:**
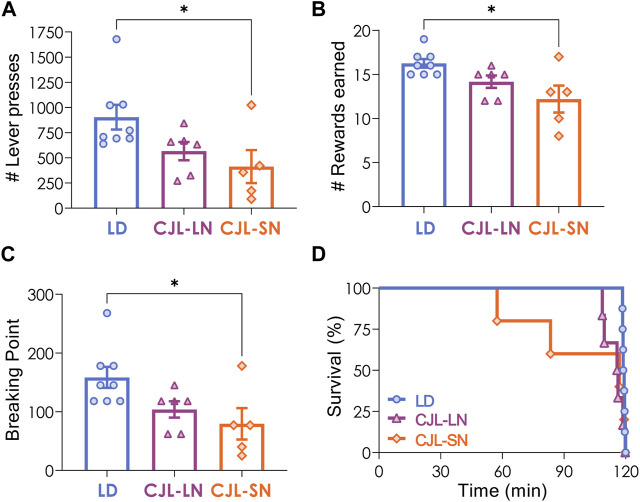
CJL decreased motivation for food reward. Animals under a 12:12 light/dark (LD) cycle or CJL were evaluated using the progressive ratio (PR) task. **(A)** Total number of lever presses (*p* = 0.0366, one-way ANOVA), **(B)** total number of rewards earned (*p* = 0.0153, one-way ANOVA), **(C)** breaking point (*p* = 0.0272, one-way ANOVA), and **(D)** percentage of animals active during the task (survival %, *p* = 0.3364, Mantel-Cox test). *p < 0.05, Tukey test. Data are expressed as mean ± S.E.M. N = 8 for LD, n = 6 for CJL-LN, n = 5 for CJL-SN (one mouse from this group was excluded for not learning the task). Mean ± S.E.M. is shown.

Mice under a 12:12 h LD cycle or CJL received 3 operant lever-press training sessions to learn the association between lever press and food reward. All animals except one from the CJL-SN group—which was excluded from further analysis of motivation behavior—met the criterion for operant lever press training (see Methods). A repeated measures two-way ANOVA indicated that animals learnt acquisition of responding along the 3 sessions (*p* < 0.0001 for time) but there was no difference in the number of total lever presses (*p* = 0.5424 for group). Motivation was then assessed using the progressive ratio (PR) task, in which animals had to make an increasing number of operant responses to obtain each successive reward. Data analysis revealed that the CJL-SN group exhibited significantly lower values of lever presses compared to the other groups (*p* = 0.0366, one-way ANOVA, Figure 2A). Furthermore, the CJL-SN group had significantly fewer rewards earned and a lower breaking point (rewards: *p* = 0.0153, one-way ANOVA, Figure 2B; breaking point: *p* = 0.0272, one-way ANOVA; Figure 2C). On the other hand, analysis of survival %—i.e., the percentage of animals active during the task—indicated that although CJL-SN presented lower survival than the other groups, this difference was not statistically significant (*p* = 0.3364, Mantel-Cox test, Figure 2D). Overall, these findings indicate a lower motivation for food reward in mice under CJL protocol evaluated during the short night.

### CJL increased anxiety and depressive-like behaviors

**FIGURE 3:**
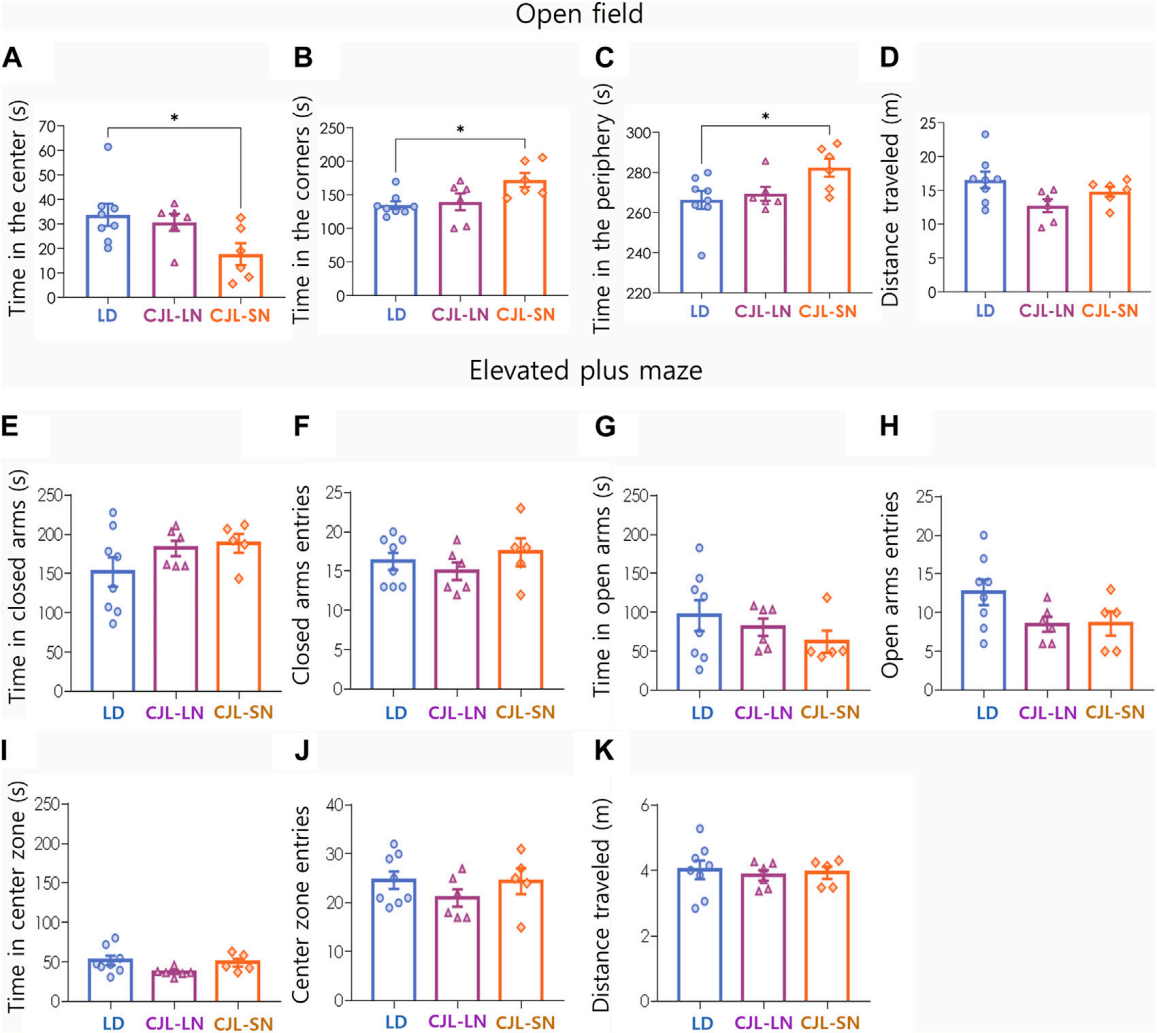
CJL increased anxiety-like behavior in the open field but not in the elevated plus maze. Upper panel **(A–D)** displays the results of the open field task. **(A)** Time in the center zone (*p* = 0.0430, one-way ANOVA). **(B)** Time in the corners (*p* = 0.0254, one-way ANOVA). **(C)** Time in the periphery (*p* = 0.0430, one-way ANOVA). **(D)** Total distance traveled (*p* = 0.3084, one-way ANOVA). *p < 0.05, Tukey test. Mean ± S.E.M. is shown. N = 8 for LD, n = 6 for CJL-LN, n = 6 for CJL-SN. Bottom panel **(E TO K)** shows the results of the elevated plus maze task. **(E)** Time spent in closed arms (*p* = 0.2284, one-way ANOVA). **(F)** Closed arms entries (*p* = 0.4840, one-way ANOVA). **(G)** Time spent in open arms (*p* = 0.2130, one-way ANOVA). **(H)** Open arms entries (*p* = 0.0960, one-way ANOVA). **(I)** Time spent in the center zone (*p* = 0.1110, one-way ANOVA). **(J)** Center zone entries (*p* = 0.3919, one-way ANOVA). **(K)** Total distance traveled (*p* = 0.3719, one-way ANOVA). Mean ± S.E.M. is shown. N = 8 for LD, n = 6 for CJL-LN, n = 5 for CJL-SN (1 outlier was detected in the latter group).

Anxiety-like behaviors were assessed using two different tests: the open field (OF) and the elevated plus maze (EPM). In the OF test, CJL mice evaluated in the short night (CJL-SN) had a significant reduction in the time spent in the center (*p* = 0.0430, one-way ANOVA, Figure 3A) and an increase in the times spent in the corners (*p* = 0.0254, one-way ANOVA, Figure 3B) and periphery (*p* = 0.0430, one-way ANOVA, Figure 3C). The total distance traveled was not affected by CJL (*p* = 0.3084, one-way ANOVA, Figure 3D). On the other hand, the CJL protocol did not have an impact on closed arm exploration (Figures 3E,F, *p* = 0.2284 and *p* = 0.4840 respectively, one-way ANOVA), open arm exploration (Figures 3G,H, *p* = 0.2130 and *p* = 0.0960 respectively, one-way ANOVA), center zone (Figures 3I,J, *p* = 0.1110 and *p* = 0.3919 respectively, one-way ANOVA) and distance traveled (Figure 3K, *p* = 0.3719, one-way ANOVA) in the EPM test. These findings indicate a mixed anxiety phenotype, with an increase in anxiety-like behavior observed specifically in the open field test, while the other behavioral task (elevated plus maze) remained unaffected by the CJL conditions.

**FIGURE 4:**
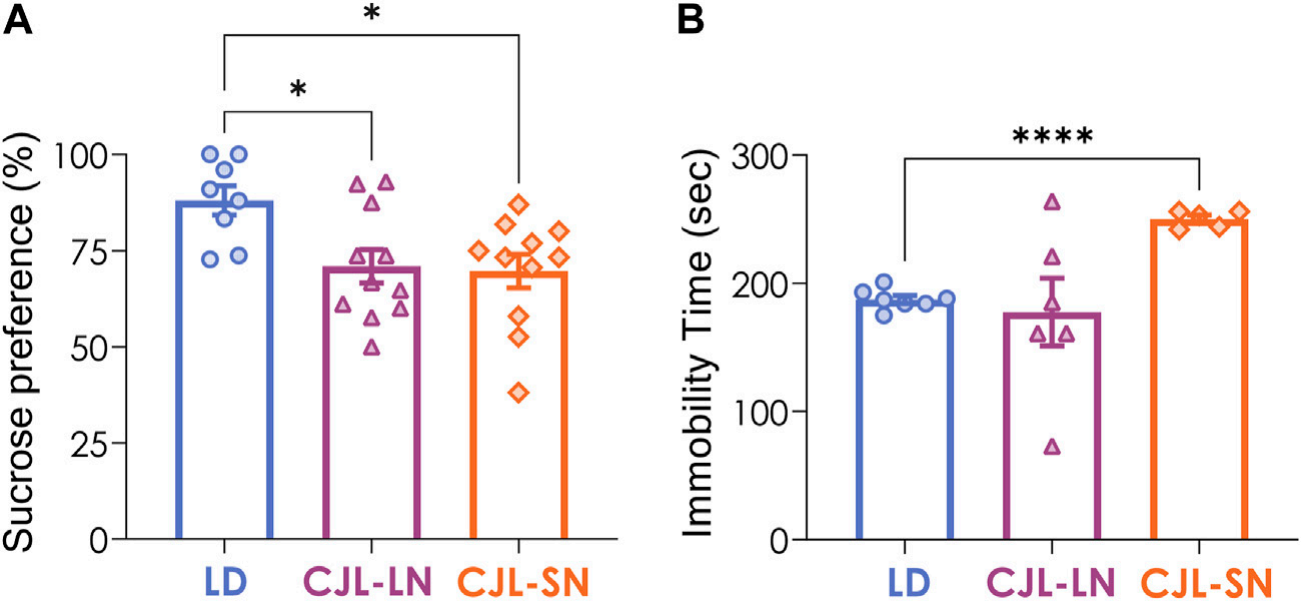
CJL increased depressive-like behaviors. **(A)** Sucrose preference test (SPT) was evaluated for anhedonia. Both CJL-LN and CJL-SN groups had lower preference for sucrose than the LD group (*p* = 0.0142, one-way ANOVA). *p < 0.05, Tukey test. N = 8 for LD, n = 12 for CJL-LN, n = 12 for CJL-SN. **(B)** The tail suspension test (TST) was used to evaluate depressive-like behavior. The CJL-SN group presented higher immobility time compared to the LD group (*p* < 0.0001, one-way ANOVA with Welch’s correction). ***p < 0.0001, Tukey test. N = 7 for LD (one outlier detected), n = 6 for CJL-LN, n = 5 for CJL-SN (one outlier detected). Mean ± S.E.M. is shown.

Anhedonia and behavioral despair represent two essential symptoms associated with depression. Anhedonia was evaluated by the sucrose preference test (SPT). During week 8 of CJL protocol, mice were provided with a two-bottle choice between 2% sucrose and water for a 24 h period. The control group exposed to a 12:12 h LD cycle exhibited a greater preference for the sucrose solution (88.1%) compared to both the CJL-LN and CJL-SN mice (70.9% and 69.7%, respectively; *p* = 0.0142, one-way ANOVA, Figure 4A). Depression was assessed by using the tail suspension test (TST). Immobility time in the TST was higher in CJL-SN mice than in the other groups (*p* = 0.0283, one-way ANOVA, Figure 4B). These data indicate that CJL increases anhedonia and a depression-like state, and that the effect on depression is more pronounced when animals are evaluated on the short night of CJL.

## Discussion

The circadian system plays a crucial role in regulating a broad spectrum of behavioral functions, including the intricate processes related to reward (Acosta et al., 2020). Chronic jet lag leads to desynchronization of circadian rhythms, causing a wide range of physiological and psychological effects (Golombek et al., 2013). In the present study, C57BL/6J male mice were subjected to circadian disruption through phase advances of 6 h every 2 days (CJL +6/2) and a battery of behavioral experiments was performed. After 8 weeks of CJL, desynchronization of activity-rest rhythms led to several behavioral impairments, including an increase in anxiety and depressive-like behavior, and decreased motivation for food reward. Similar results as here were found in rats, such as anhedonia and increased anxiety, using an advancing CJL protocol that also decreased hippocampal neurogenesis and impaired learning and memory (Herzog et al., 2017; Horsey et al., 2020). In addition, unlike previous results from the laboratory (Casiraghi et al., 2016; Trebucq et al., 2023), CJL did not increase body weight. Other works using CJL protocols also failed to report changes in body weight (Preuss et al., 2008; Chen et al., 2021), nevertheless, internal desynchronization can still take place at both the organ and the molecular levels (Wolff et al., 2013; Siddique et al., 2022).

The lack of a stable phase of entrainment of the SCN clock with the LD cycle generates desynchronization of outputs controlling key circadian organizers, as activity/feeding—rest/fasting behavioral rhythms, hypothalamic nuclei controlling sleep/arousal, temperature, energy metabolism, hypothalamic-pituitary-adrenal (HPA) neuroendocrine axis controlling stress, etc. Uncoupling the circadian phase of activity/feeding-rest/fasting rhythms from central and peripheral control of energy metabolism increase the risk of metabolic alterations (Trebucq et al., 2023), which can be considered to increase the risk for depressive states (Gu et al., 2021). Similar CJL protocols show disruption of daily homeostasis in brain energy metabolism (i.e., glucose disposal/usage), increasing mood disorders without affecting sleep duration (Gao et al., 2020; Siddique et al., 2022).

Most of the behaviors evaluated in the present manuscript exhibit circadian rhythms in rodents. In the OF test, mice locomotion in the dark phase (i.e., their active phase) is greater than in the light phase (Kopp, 2001). Smaller exploratory behavior in the light phase was also observed for the EPM in rats (Andrade et al., 2003). We demonstrated that motivation for food reward in mice presents higher levels during the night compared to the day (Acosta et al., 2020). For this reason, in order to observe an effect of CJL on mood-related behaviors, the night portion of the LD cycle was chosen to perform the behavioral experiments. Under CJL, the activity-rest rhythm splits into two components, one which is driven by the light schedule having a period of 21 h, and a “free-running” component, with a spontaneous circadian period (Casiraghi et al., 2012). This behavioral pattern corresponds to specific activities and outputs of two SCN regions (as under a T21 cycle, de la Iglesia, et al., 2004), the dorsomedial driving the circadian gate for sleep and core temperature, while ventrolateral SCN drives the homeostatic sleep pressure (Cambras et al., 2007). In the present work, mice under CJL were tested at the coincident activity phases (dark) of the two period components, where main activity bouts are present (as can be seen in the actograms in Supplementary Figure S2). These short and long nights can be considered as the outputs of SCN circadian oscillators, each with their own amplitude values. Our hypothesis is that a decrease of such amplitude in the mood-related behaviors is due to a chronic effect of the ChrA^6/2^ protocol. Also, partial entrainment of activity-rest due to CJL, generates a circadian component with a chronically delayed phase related to the LD cycle (analogous to the evening chronotype) which links to depression in humans (Zou et al., 2022).

In addition, several features of the CJL light protocol can be considered. First, the reduction of photoperiod (light duration per 24 h), was shown to increase mood disorders and depression-like behaviors related to decreasing brain serotonin levels and glucose sensitivity at serotonergic systems (Otsuka et al., 2014). Also, exposure to light at subjective night (so-called light-at-night, or LAN protocols) when mice are behaviorally active, generates depressive-like behaviors (An et al., 2020; Walker et al., 2020) without affecting timing or quality of sleep (Borniger et al., 2013). These effects of LAN on mood and depression are demonstrated to be non-circadian, involving intrinsically photosensitive retinal ganglion cells expressing melanopsin (LeGates et al., 2012) connecting to the perihabenular thalamic nucleus (Fernandez et al., 2018) and the nucleus accumbens (An et al., 2020). Moreover, dysregulation of the serotonergic system by stress mechanisms can be taken as an effector between CJL and mood disorders (Daut and Fonken, 2019). Disruption of circadian rhythms can affect serotonin functions related to neurological disorders, such as depression, bipolar, and obsessive-compulsive disorders (McClung, 2007). Indeed, transcriptional profiling in mice under CJL shows impairments in genes regulating serotonergic transmission at the nucleus accumbens and prefrontal cortex (Siddique et al., 2022).

Our results demonstrate decreased motivation and increased anxiety levels observed specifically in the CJL-SN group compared to CJL-LN and LD groups. Under our JLC +6/2 protocol, the activity-rest pattern can be taken as the expression of two circadian oscillators, one that is entrained to the LD cycle with a period of 21 h, and other that free-runs with a period larger than 24 h (see Casiraghi et al., 2012). A possible framework to explain what is observed for changes in mood-related behaviors in the CJL-SN group is that mice were tested at the coincidence of activity phases of such oscillators, when they are likely expressing their “subjective circadian night” (de la Iglesia et al., 2004). Thus, performance in mood-related behaviors in the CJL-LN was similar to values found for the night under normal LD, where the long night occurred at the beginning of such coincidence. A decrease in performance at the short night, could be related to an inertial effect of the two oscillators beginning to be separated one cycle later, by the pushing, chronic effect of light acting on the light entrained oscillator.

Some limitations of the present study include the lack of different time points for assessing behavior or adding an antidepressant treatment, as well as the use of only male mice. For these reasons, we have not fully characterized the exact mechanism by which CJL affects mood-related behaviors. Indeed, chronic circadian disruption may affect clock regulated cellular processes leading to neurodegerative brain disorders (Logan and McClung, 2019) including its effects on mood-related behaviors (chronic effects). Although, testing the mood-related behaviors at the coincident activity phases of the short and long period components strengthens the hypothesis that CJL affects mood through its chronic effects; but because the behaviors were tested at a single time point a day, we cannot completely rule out the possibility that the increased signs of depression do not arise only from CJL induced alteration in the phase of these mood-related behavioral rhythms. Testing mood-related behaviors at several time points throughout the day would be necessary to certainly rule out the phase alteration possibility. For this reason, a limitation of the present study is that it cannot unambiguously distinguish between these two possibilities: 1) altered phase of the circadian rhythm of mood-related behavior, and 2) a general worsening of depression indicators. These additional factors may be worth evaluating in the future.

In conclusion, a central role of the circadian clock is to entrain the organism to the external environment via photic (the light/dark cycle) or non-photic (i.e., food availability) cues (Golombek and Rosenstein, 2010). In situations where the circadian system is unable to maintain its synchronization with the external cycles, such as CJL or shift work, it results in a state of circadian disruption. The relationship between circadian disruption and mental disorders has been studied to the point that a clinical phenotype called “circadian depression” was proposed, which is interpreted as a cross-cutting phenotype, including diagnoses of depression, bipolar syndrome, anxiety, and psychosis, related with irregular sleep patterns (Carpenter, et al., 2021).

The findings of the present work provide evidence supporting the hypothesis that the chronic disruption of the circadian organization can contribute to or worsen abnormalities in mood-related behaviors, increasing the likelihood of reduced motivation and depression. In particular, our understanding of the relationship between the circadian system and depression remains incomplete, and alterations in sleep/wake, core body temperature, neuroendocrine and neurotransmitter rhythms are usually taken as consequences, instead of causes, of depression (Bunney and Bunney, 2000). Our results suggest that circadian disruption might also be taken into account and, moreover, circadian therapies alleviating depression should also be considered. Moreover, our results are important for implementing therapies in situations where the circadian system is unable to maintain its synchronization with the external world, such as time zone changes or shift work.

## Data Availability

The raw data supporting the conclusion of this article will be made available by the authors, without undue reservation.
