# Supplementary material for: Chronic jet lag reduces motivation and affects other mood-related behaviors in male mice
Source: Front Physiol. 2023 Sep 6;14:1225134. doi: 10.3389/fphys.2023.1225134 (PMC10511878; doi:10.3389/fphys.2023.1225134)
Supplement: Supplementary file 1 [file Table1.docx]

**Acosta et al., 2023, Chronic jet lag reduces motivation and affects other mood-related behaviors in male mice**

**Supplementary material**


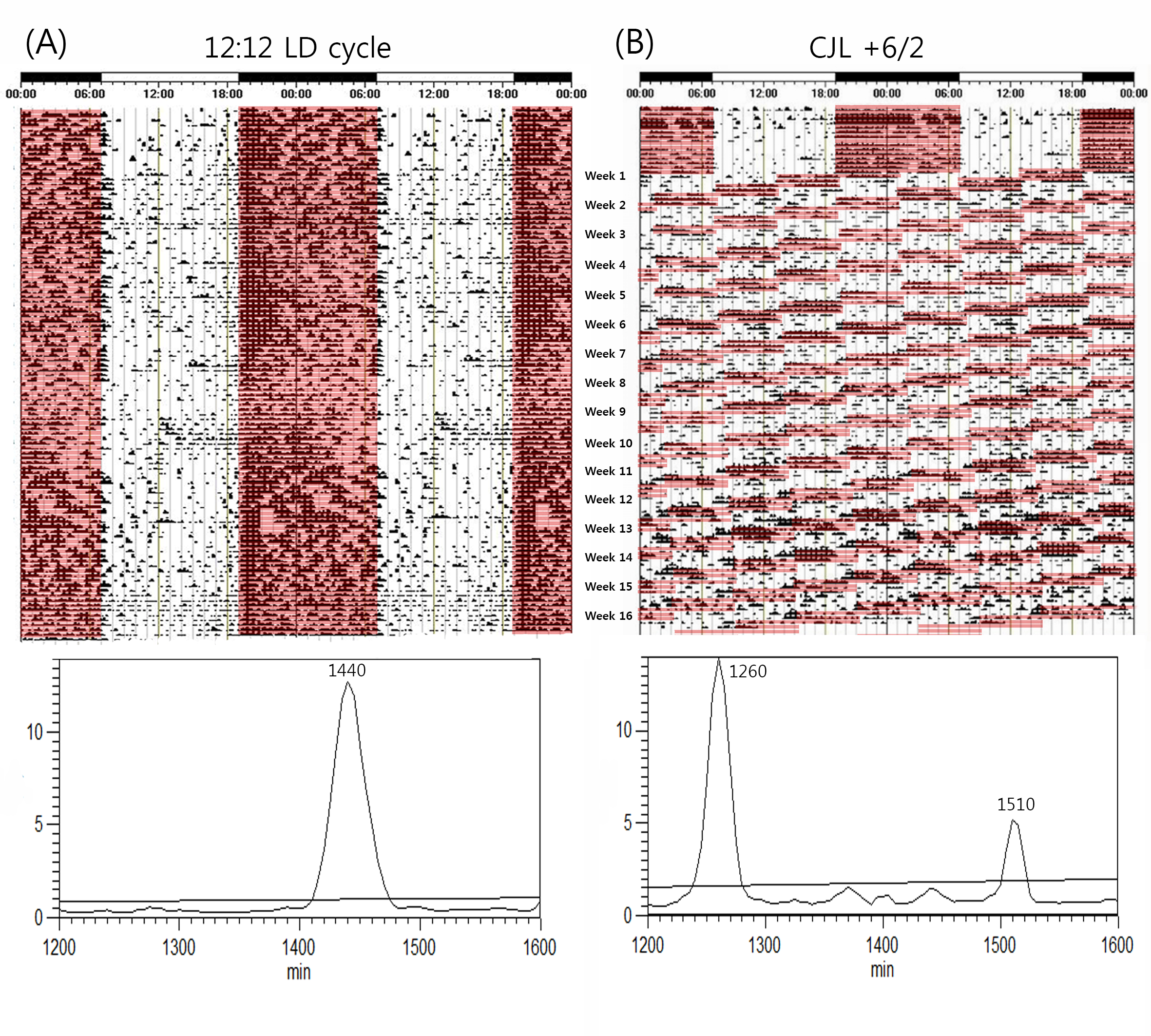


**Supplementary figure 1. Locomotor activity indicates internal desynchronization in CJL.** Representative double-plot actograms and Sokolove-Bushell periodograms of male mice under (A) a 12:12 LD cycle, or (B) a protocol of CJL +6/2. The white and black bars at the top of the actogram indicate light (12-h) and darkness (12-h), respectively, while the pink shadow indicates the dark portion of the LD cycle. The Y-axis represents consecutive days, while time is plotted in hours in the X-axis. In (B), mice were maintained for 15 days under a 12-12 LD cycle before the beginning of CJL protocol. An oblique line in the periodogram indicates a significance level of p = 0.05. Periodogram analysis shows a unique period component around 24-h (1440 min) in (A) and two components in (B): a short-period component around 21-h (1260 min, following the LD schedule), and a second component with a period greater than 24-h (1510 min), thus indicating internal desynchronization (Casiraghi et al., 2012).

**Supplementary table 1. Period length (minutes) of short and long components in mice under CJL**

| **CJL**  **mouse**  **ID** | **Short peak period (min)** | **Long peak period (min)** | |
| --- | --- | --- | --- |
| **Mouse 1** | **1260** | | **1514** |
| **Mouse 2** | **1260** | | **1512** |
| **Mouse 3** | **1260** | | **1491** |
| **Mouse 4** | **1260** | | **1511** |
| **Mouse 5** | **1260** | | **1512** |
| **Mouse 6** | **1260** | | **1511** |
| **Mouse 7** | **1260** | | **1510** |
| **Mouse 8** | **1260** | | **1510** |
| **Mouse 9** | **1260** | | **1515** |
| **Mouse 10** | **1260** | **1512** | |
| **Mouse 11** | **1260** | **1516** | |
| **Mouse 12** | **1260** | | **1513** |


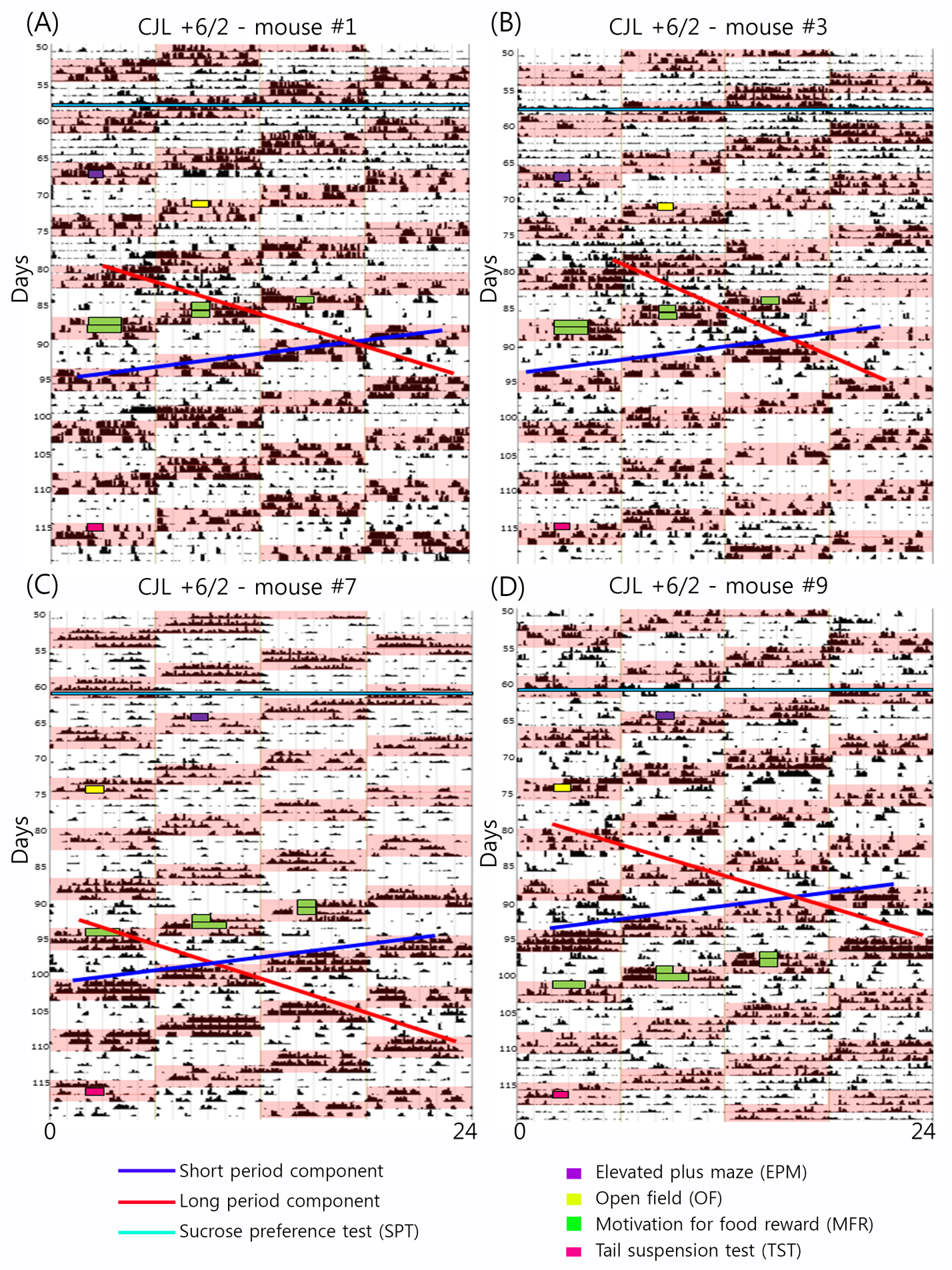


**Supplementary figure 2. Representative actograms of mice under CJL.** (A) to (D) display representative single-plot actograms of male mice under CJL +6/2 protocol. The pink shadow indicates the dark portion of the LD cycle. The Y-axis represents consecutive days, while time is plotted in hours in the X-axis. Behavioral experiments are marked with different colors. The blue and red lines indicate the short and long period components, respectively.


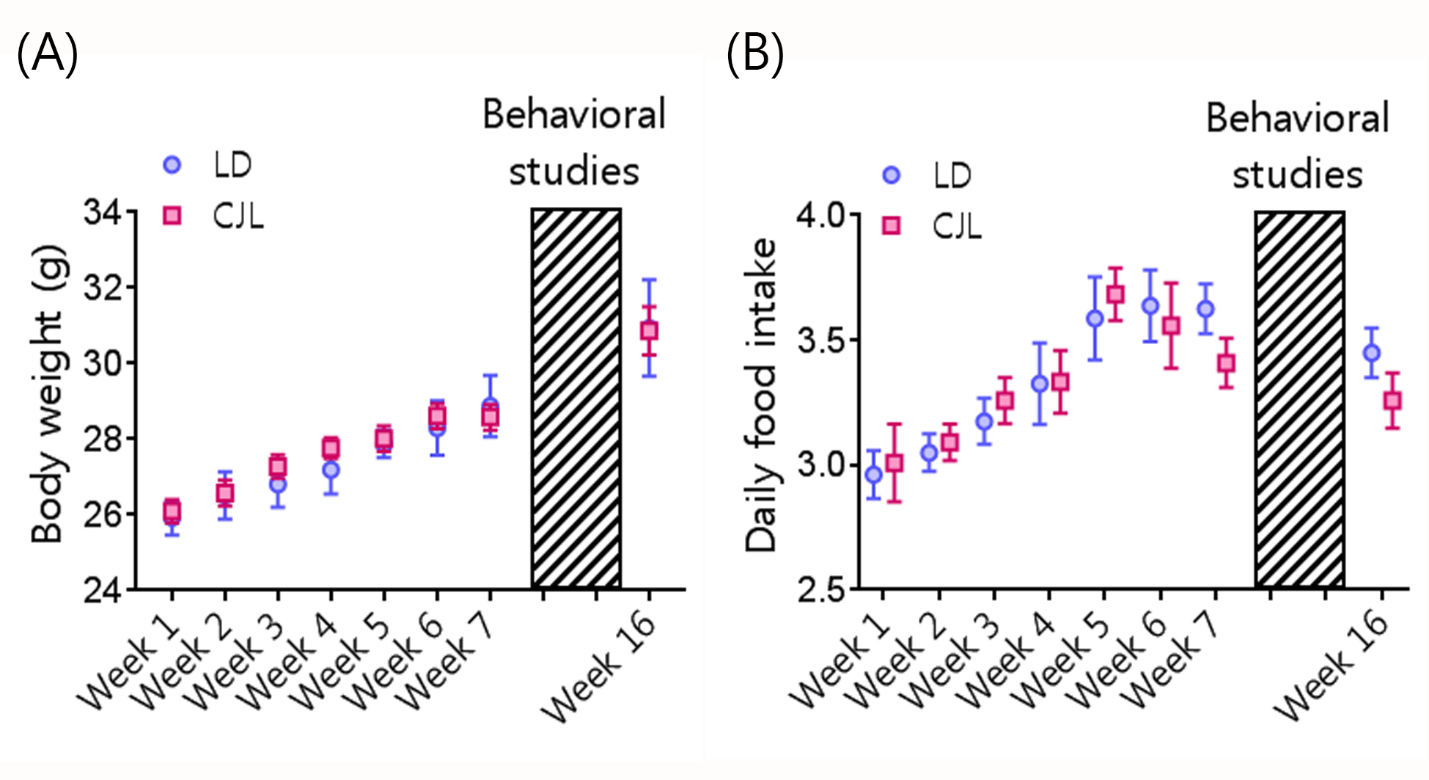


**Supplementary figure 3. CJL does not affect body weight and daily food intake.** (A) Mice were weighted once per week until the beginning of behavioral studies (week 8 of CJL). (B) Daily food intake was calculated. Animals were not disrupted for body weight and food intake during behavioral experiments except when required (e.g., for caloric restriction during motivation). There was no group difference in body weight or food intake along the experiment (Body weight: p = 0.6633; daily food intake: p = 0.7428, two-way repeated measures ANOVA). Data are shown as mean ± S.E.M. N=8 for LD, N=12 for CJL.
